# Spanlastics as a Potential Platform for Enhancing the Brain Delivery of Flibanserin: In Vitro Response-Surface Optimization and In Vivo Pharmacokinetics Assessment

**DOI:** 10.3390/pharmaceutics14122627

**Published:** 2022-11-28

**Authors:** Waleed S. Alharbi, Rawan H. Hareeri, Mohammed Bazuhair, Mohamed A. Alfaleh, Nabil A. Alhakamy, Usama A. Fahmy, Abdullah A. Alamoudi, Shaimaa M. Badr-Eldin, Osama A. Ahmed, Shareefa A. AlGhamdi, Marianne J. Naguib

**Affiliations:** 1 Department of Pharmaceutics, Faculty of Pharmacy, King Abdulaziz University, Jeddah 21589, Saudi Arabia; 2 Department of Pharmacology and Toxicology, Faculty of Pharmacy, King Abdulaziz University, Jeddah 21589, Saudi Arabia; 3 Department of Pharmacology, Faculty of Medicine, King Abdulaziz University, Jeddah 21589, Saudi Arabia; 4 Center of Excellence for Drug Research and Pharmaceutical Industries, King Abdulaziz University, Jeddah 21589, Saudi Arabia; 5 Mohamed Saeed Tamer Chair for Pharmaceutical Industries, King Abdulaziz University, Jeddah 21589, Saudi Arabia; 6 Department of Pharmaceutics and Industrial Pharmacy, Faculty of Pharmacy, Cairo University, Giza 11562, Egypt; 7 Cancer and Mutagenesis Unit, Department of Biochemistry, Faculty of Science, King Fahd Medical Research Center, King Abdulaziz University, Jeddah 21589, Saudi Arabia

**Keywords:** spanlastics, flibanserin, trans-nasal, brain levels, pharmacokinetics, response surface, D-optimal design

## Abstract

Flibanserin was licensed by the United States Food and Drug Administration (FDA) as an oral non-hormonal therapy for pre-menopausal women with inhibited sexual desire disorder. However, it suffers from susceptibility to first-pass metabolism in the liver, low aqueous solubility, and degradation in the acidic stomach environment. Such hurdles result in a limited oral bioavailability of 33%. Thus, the aim of the study was to utilize the principles of nanotechnology and the benefits of an intranasal route of administration to develop a formulation that could bypass these drawbacks. A response-surface randomized D-optimal strategy was used for the formulation of flibanserin spanlastics (SPLs) with reduced size and increased absolute zeta potential. Two numerical factors were studied, namely the Span 60: edge activator ratio (*w*/*w*) and sonication time (min), in addition to one categorical factor that deals with the type of edge activator. Particle size (nm) and zeta potential (mV) were studied as responses. A mathematical optimization method was implemented for predicting the optimized levels of the variables. The optimized formulation was prepared using a Span: sodium deoxycholate ratio of 8:2 *w*/*w*; a sonication time of 5 min showed particle sizes of 129.70 nm and a zeta potential of −33.17 mV. Further in vivo assessment following intranasal administration in rats showed boosted plasma and brain levels, with 2.11- and 2.23-fold increases (respectively) compared to raw FLB. The aforementioned results imply that the proposed spanlastics could be regarded as efficient drug carriers for the trans-nasal delivery of drugs to the brain.

## 1. Introduction

Flibanserin (FLB) was licensed by the United States Food and Drug Administration (FDA) in 2015 as a non-hormonal oral therapy prescribed for pre-menopausal females suffering from a hyposexuality disorder [1,2]. It acts as a full agonist of the serotonin 1A receptor (known as the 5-HT1A receptor) and an antagonist to the serotonin receptor subtype 2A (5-HT2A receptors), thus decreasing serotonin secretion, while increasing dopamine and norepinephrine levels. Its impacts on these neurotransmitters are important for sexual excitation, arousal, and desire enhancement, all of which are important areas of holistic health and wellbeing [3,4]. However, FLB’s restricted oral bioavailability, which is about 33%, represents a hurdle for its clinical use [5]. Its low bioavailability may be attributed to the significant first-pass metabolism in the liver, its low water solubility, and its degradation in the environment of the stomach [4,6]. Therefore, it would be a clinically significant solution to modify the formulation and use a different route of administration for this drug.

Over the last few decades, the exploitation of nanotechnology in the delivery of drugs has been widely investigated as a new technique for overcoming weak bioavailability and delivering medications to the target site (mainly CNS) of different pharmaceutical medications. Such systems include polymeric nanoparticles and nanovesicular systems, which can protect active compounds against enzymatic and chemical degradation, as well as extend the duration during which medications are diffused in the bloodstream [7,8].

Spanlastics (SPLs) are elastic nanovesicle systems invented by Kakkar and Kaur [9]. They are made up of a nonionic surfactant, ethanol, and an edge activator (EA) [8]. Both hydrophilic and hydrophobic medications can be delivered via spanlastics, which are encased in a compartment formed by interior hydrophilic and outer lipid layers [10]. Spanlastics are non-immunogenic vesicular carriers that are safe, biodegradable, and non-toxic. Many studies have shown that spanlastics can significantly improve therapeutic efficacy, increase drug bioavailability, improve patient compliance, and minimize adverse effects [11]. Furthermore, because of their elasticity, these vesicular vehicles have an advantage over liposomes in that they are more chemically stable, and have further benefits in comparison to niosomal colloidal delivery systems [9]. Additionally, the presence of an edge activator acts as a disrupting element in the lipid membrane of the vesicles and enhances the permeability of the nano-sized vesicles, increasingly the deformability across biological membranes, which are all also recognized benefits of the elasticity of spanlastic vesicles [11].

There has recently been a surge in interest in the use of spanlastics to improve drug delivery. Al-mahallawi et al. formulated nano-spanlastics that have the potential to enhance ciprofloxacin trans-tympanic delivery [12]. Another study successfully prepared a spanlastic carrier encapsulated with clotrimazole, and showed that this formulation can be effective for the ocular delivery of clotrimazole with improved antifungal activity in the treatment of fungal keratitis [13]. In addition, terbinafine hydrochloride-loaded spanlastic nanovesicles improved penetration of the drug through the nails, and confirmed that spanlastics show promise for trans-ungual terbinafine hydrochloride delivery [14]. Spanlastics have been utilized to improve the topical delivery of fenoprofen calcium in the treatment of arthritis, allowing for both sustained and increased anti-inflammatory activity [15].

Intranasal administration has gained attention for the distinctive ability of this route to deliver drugs into the brain, following the recognized pathway of trigeminal nerve and the olfactory bulb route. The advantages of intra-nasal administration include eliminating the first-pass metabolism, decreasing drug degradation by gastric fluids, being a non-invasive technique, reducing infrequent dosing, reducing systemic side effects, and fast drug absorption due to the presence of a highly vascularized structure [16]. Because of its advantages, intra-nasal administration has recently become the preferred technique of medication delivery to the CNS. Intra-nasal delivery as a route of drug administration does have the added feature of delivering drugs to the brain by tracking the trigeminal nerve network through the upper area in the nose, starting from the olfactory nerve, where it is conveyed promptly from the nasal cavity (olfactory nerve) to the CNS (olfactory bulb) [17]. Interestingly, some studies have discovered that spanlastics have the ability to deliver specific medicines to the brain via an intra-nasal route of administration. For example, increased drug penetration across the nasal membrane was seen in zolmitriptan-loaded spanlastic formulations, affirming the promising impact of the intra-nasal dosing ability for brain delivery [11]. Moreover, granisetron hydrochloride spanlastic gel formation appears to promote the brain bioavailability of the carried drug, offering an increased level of treatments in the brain [18,19,20]. Yassin et al. [18] used spanlastics to improve carbamazepine delivery to the CNS (brain) via the intra-nasal method.

Hence, building on previous research, this study aims to combine the benefits of spanlastics delivery, the leverage offered by the intranasal route of administration, and the ability of nanotechnology to enhance FLB brain delivery.

## 2. Materials and Methods

### 2.1. Materials

The active ingredient of flibanserin was procured from Qingdao-Sigma Chemical Co., Ltd. (Qingdao, China). The other materials—Span^®^ 60, Tween^®^ 80, sodium deoxycolate (SDC), polyvinyl alcohol (PVA), D-α-tocopheryl polyethylene glycol succinate (TPGS), and solvents—were procured from Sigma-Aldrich Chemie GmbH (Taufkirchen, Germany).

### 2.2. Methodology

#### 2.2.1. Experimental Design

To optimize FLB-SPLs with desirable pharmaceutical characteristics, response-surface-randomized D-optimal design was employed. This approach enables the assessment of the influence of both numeric and categorical factors on responses. In this study, two numeric factors were investigated: the span to edge activator weight ratio (Span: EA, X_1_, *w*/*w*) and the sonication time (ST, X_2_, min). Additionally, one categorical factor, edge activator type (EA, X_3_), was investigated. The independent variables were selected based on the literature, while their ranges were chosen based on preliminary studies performed in our lab. Particle size (PS, Y_1_, nm) and zeta potential (ZP, Y_2_, mV) were considered as responses. The levels of the investigated variables, as well as the objective targets for the responses, are listed in Table 1. Design Expert^®^ software (Version 11.0, Stat- Ease Inc., Minneapolis, MN, USA) was used to generate 18 design points. The combination of variables in each design point is displayed in Table 2. The responses were investigated statistically using analysis of variance (ANOVA) to elucidate the influence of the variables on the responses at *p* < 0.05. Linear plots were constructed to display the individual effects of the investigated variables.

#### 2.2.2. Preparation of FLB-SPLs

The ethanol injection method was utilized to prepare the FLB-SPLs [21]. Absolute ethanol (10 mL) was used to dissolve FLB (20 mg) and specified amounts of FLB and Span. Thereafter, the ethanolic solution was quickly inserted into a solution of 10 mL edge activator in water, which was made at a temperature of 65 °C. Calculations of the Span and edge activator quantities were based on their ratios (as per the experimental design) with total amounts of 500 mg. The mixture was stirred at 1000 rpm for 45 min to allow for organic solvent evaporation. After volume adjustment to 10 mL, a dispersion was produced, which was then subjected to ultrasonication for a time ranging from 0–5 s (according to the design).

In addition, a raw FLB suspension was prepared for in vivo study by suspending FLB in a 0.5% sodium carboxy methyl cellulose aqueous dispersion, which was stirred at 400 rpm using a magnetic stirrer.

#### 2.2.3. Characterization FLB-SPLs

The prepared FLB-SPLs were characterized regarding PS (z-average, intensity weighted mean hydrodynamic size) and ZP using a Zetasizer Nano ZSP (Malvern Panalytical Ltd., Malvern, UK). The samples were properly diluted prior to the measurements. The obtained mean results (five runs each) were presented.

#### 2.2.4. Optimization of FLB-SPLs

Mathematical optimization and the desirability function methodology were used to predict the optimized levels of the independent variables that could achieve the lowest particle size and the highest absolute zeta potential.

##### Characterization of the Optimized FLB-SPLs

The optimized FLB-SPLs entrapment efficiency (EE%) was investigated by indirect assessment. In brief, the optimized FLB-SPLs were diluted with water, then centrifuged (400,000× *g*, 45 min). The aqueous supernatant was collected and analyzed for FLB content using the HPLC method developed in the laboratory with acetonitrile/water (9:1), containing 0.1% formic acid, and analyzed at 260 nm, as previously reported [6]. The EE% of FLB in the optimized FLB-SPLs was determined utilizing Equation (1):

(1) $$\mathrm{EE}\;\%\;\mathrm{of}\;\mathrm{FLB}\;\mathrm{in}\;\mathrm{FLB}-\mathrm{SPLs}=\frac{FLB\;amount\;originally\;added-FLB\;amount\;in\;the\;supernatent}{FLB\;amount\;originally\;added}\times 100$$

The optimized FLB-SPLs were also explored using a transmission electron microscope (TEM), JEOL GEM-1010 (JEOL Ltd., Akishima, Tokyo, Japan) at the Regional Center for Mycology and Biotechnology (Al-Azhar University, Cairo, Egypt). One drop of the optimized FLB-SPLs (dispersed in water) was distributed on a grid (carbon-coated) and dried. After that, 1% phosphotungstic acid (negative staining) was added to the optimized FLB-SPLs, dried, then visualized.

#### 2.2.5. In Vivo Pharmacokinetic Study

##### Animals

Male Wistar rats (200 and 250 g) were utilized to estimate the FLB’s pharmacokinetic parameters after the intra-nasal administration of the optimized flibanserin-spanlastic FLB-SPLs formula, in comparison to rats receiving control raw FLB suspension. The Research Ethics Committee reviewed and approved the study protocol (reference number PH-124-41). Rats were kept under standard conditions of temperature and humidity (25 °C ± 0.5 and ≈ 65%, respectively) in light-controlled cages, with alternate day and night cycles (12 h each) [22]. Animals were granted free access to water and a standard rat diet, according to the Guide for the Care and Use of Laboratory Animals [23].

##### Study Design

On the first day of the study, the 48 enrolled rats were randomly allocated into two groups (24/group). Group I received the optimized FLB spanlastic formula, while group II was administered the raw FLB suspension in a dose of 10 mg/Kg. The administration in both groups was conducted intranasally. The rats were held from the back in an inclined position for intranasal delivery, and treatments were delivered using a micropipette (200 µL) fixed with a low-density polyethylene tube of 0.1 mm internal diameter at the delivery site. The treatment was carried out delicately so that the animals could breathe throughout the preparation [24]. At specified time intervals (0.25, 0.5, 1, 2, 4, 6, 8, and 24 h), three rats from each group were sacrificed by cervical dislocation; the brain of each rat was then obtained, washed with phosphate buffer saline (PBS, pH 7.4), weighed, and diluted with 3 × its weight of PBS. Each brain was then homogenized (T 25 digital ULTRA-TURRAX^®^, Staufen, Germany) separately at 10,000 rpm for 10 min. Samples of blood were also collected form the dissected rats. Plasma was separated from the blood samples by centrifuging at 4000 rpm for 20 min. The samples of the homogenized brain and separated plasma were all kept at −80 °C until analysis.

##### FLB Assay

FLB concentrations were measured in the collected samples utilizing the LC/MS/MS technique via an in-house quantitation technique [4]. In brief, each sample (300 µL) was mixed with 100 µL of flibanserin-d as an internal standard at a concentration of 500 ng/mL, and 4 mL of ethyl acetate. The mixtures were then vortexed and centrifuged. Afterwards, the organic layer was dried under vacuum and the residues were then reconstituted with 300 µL of the mobile phase containing acetonitrile and water, with 0.1% formic acid in an 8:2 ratio. The samples were injected into a Shimadzu LC/MS/MS (Tokyo, Japan) equipped with a Sunfire column (C18, 4.6 × 50 mm, Waters Corporation, Milford, MA, USA) and a mobile phase flowing at 0.98 mL/min in an isocratic elution approach. The apparatus functioned with a scan speed of 10 Da/s and a dwell of 500 ms. The precursor-to-product ion ratios were 391.15/161 and 395.14/165 for FLB and FLB-d, respectively. A linear curve was obtained (R^2^ = 0.999) with lower and upper limits of detection of at 2, 800 ng/mL, respectively.

##### Analysis of Pharmacokinetic Parameters

The key pharmacokinetic parameters were calculated from the in vivo results utilizing non-compartmental pharmacokinetic analysis in Kinetica^®^ software (Version 5, Thermo Fisher Scientific Inc., Waltham, MA, USA). These parameters included the FLB maximum plasma concentration (C_max_), its corresponding time (T_max_), the area under the curve from zero to the last sampling point (AUC_0–24_), and the area under the curve from zero to infinity (AUC_0–∞_) in both plasma [4] and brain samples [25]. The following calculation was used to evaluate the relative bioavailability in comparison to the raw FLB powder:

(2) $$Relative\;bioavailability\;\left(RB\right)=\frac{AUC_{0-\infty \;}\;\left(TEST\right)}{AUC_{0-\infty \;}\left(reference\right)}\times 100\;$$

The findings were presented as the mean of 3 rats ± SD. Data were checked for normality using the Kolmogorov–Smirnov test. Statistical analysis of the calculated pharmacokinetic parameters was performed using the Student’s *t* test (for parametric data) or Mann–Whitney (for non-parametric data) at a *p*-value < 0.05 using SPSS^®^ 23.0 (IBM, Chicago, IL, USA).

## 3. Results and Discussion

The rationale for considering size and charge as responses and the selected target for each response can be explained by their important roles in brain delivery and physical stability, respectively. It is reported that one of the important factors that might affect medication penetration via the BBB is the size of the delivery system; nano-sized systems have more permeation power. Accordingly, reducing the size increases the surface area, which affects how well drugs are [26]. Furthermore, the size could influence reticuloendothelial-system-mediated clearance, where nanosized systems may avoid immune system capture [27]. Regarding the surface charge, it is well documented that the charge reflects the physical stability of the dispersions, where high surface charges provide electrostatic repelling forces that inhibit the coalescence and agglomeration of particles. Typically, stable systems are nano-formulations with ZP values of approximately or more than 30 mV [28,29,30]. On this basis, the study aimed to minimize particle size and maximize zeta potential

### 3.1. Model Fit Statistics

The data of each response was subjected to fit statistics analysis to determine the best-fitting polynomial model that could express the relation between this response and the investigated variables. The suggested model is based on maximized R^2^. As per the fit statistics results, the suggested model for both responses was the linear one, with R^2^ of 0.9197 and 0.9583 for PS and ZP, respectively. The predicted and adjusted R^2^ for PS were 0.8837 and 0.8113, while those for ZP were 0.9409 and 0.9045, respectively. The reasonable coincidence of the predicted and adjusted R^2^, evidenced by the comparatively low difference of less than 0.2 for both responses, highlights the model’s suitability. Furthermore, the adequate precision values of PS and ZP were 16.17 and 17.92, respectively, where values higher than 4 indicate an appropriate signal to noise ratio. Accordingly, the linear model is considered appropriate for navigation of the experimental design space.

### 3.2. Diagnostic Analysis

**Figure 1:**
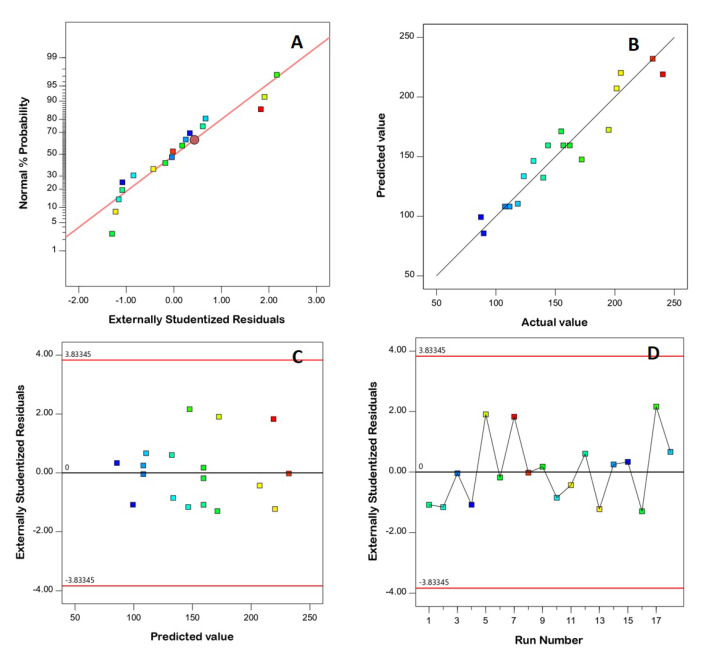
Diagnostic plots for particle size of FLB-SPLs. (**A**) Normal probability plot; (**B**) predicted vs. actual values plot; (**C**) studentized residuals vs. predicted values plot; (**D**) externally studentized residuals vs. run number plot. Abbreviations: FLB, flibanserin; SPL, spanlastics.

**Figure 2:**
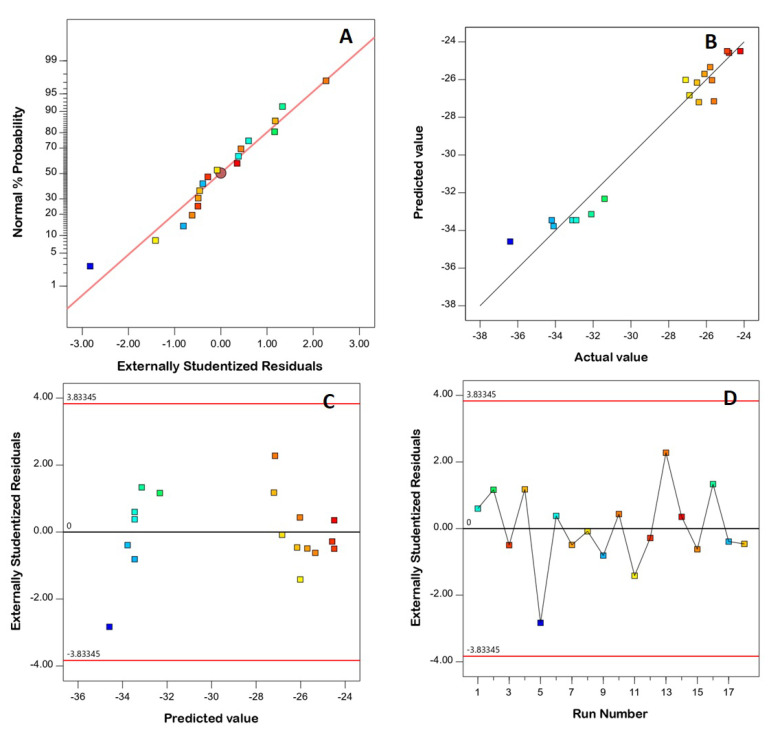
Diagnostic plots for zeta potential of FLB-SPLs. (**A**) Normal probability plot; (**B**) predicted vs. actual values plot; (**C**) studentized residuals vs. predicted values plot; (**D**) externally studentized residuals vs. run number plot. Abbreviations: FLB, flibanserin; SPL, spanlastics.

Diagnostic analysis was conducted to assess how both the PS and ZP data fit to the linear model, and diagnostic plots were generated. The linear pattern shown in the normal probability plots of residuals (Figure 1A and Figure 2A) indicates the normal distribution of residuals, and therefore the absence of the need for data transformation. The lack of a requirement for transformation is supported by the maximum-to-minimum measured responses, where a ratio higher than 10 shows the necessity of transformation, while the power transformation has little effect for ratios of less than 3. The good linearity observed in Figure 1B and Figure 2B (displaying the predicted versus actual responses) proves the good correlation between the measured and anticipated values, thus confirming the model’s validity [31]. Furthermore, Figure 1C and Figure 2C demonstrate the residual vs. run plots, and Figure 1D and Figure 2D demonstrate the residual vs. run plots, showing randomly scattered points within the limits, indicating the absence of a constant error or any lurking variable that could influence either of the responses [6,31].

### 3.3. Statistical Analysis for the Influence of Variables on PS (Y_1_)

**Figure 3:**
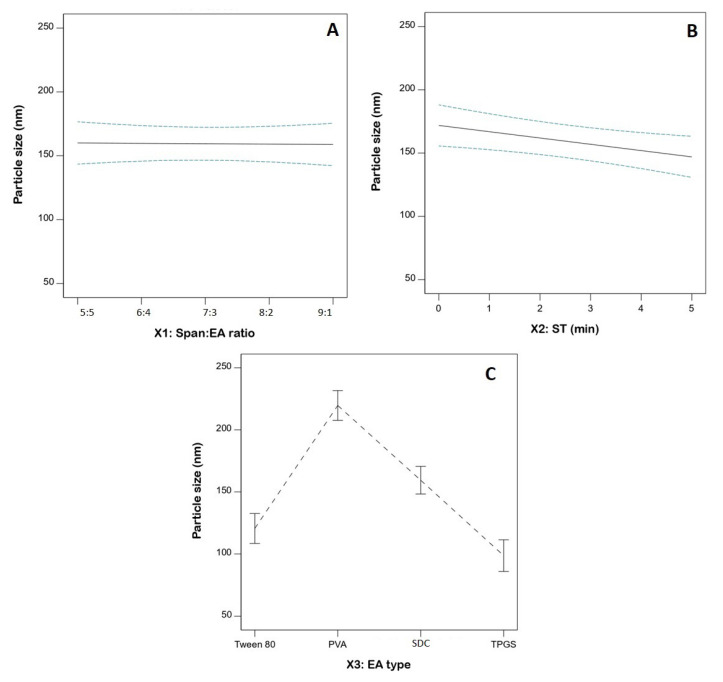
Linear plots showing individual effects on FLB-SPLs’ size. (**A**) Span: EA ratio; (**B**) ST; (**C**) EA type. Abbreviations: FLB, flibanserin; SPL, spanlastics; EA, edge activator; ST, sonication time; PVA, polyvinyl alcohol; SDC, sodium deoxycholate; TPGS, D-α-tocopheryl polyethylene glycol succinate.

PS is one of the key influential characteristics of delivery systems that can affect blood–brain barrier penetration, with clearance mediated by the reticuloendothelial system. Minimizing PS results in increased surface area, with consequently increased permeability. Furthermore, reduced PS aids in avoiding clearance by the immune system [18,19]. The average measured PS of the FLB-SPLs ranged from 87.4 ± 1.9 to 240.3 ± 6.9 nm (Table 2). The nano-size range is beneficial for boosting trans-nasal drug delivery to the brain. Homogenous and uniform distribution is evidenced by the comparatively small standard deviation. ANOVA for PS is presented in Table 3. The computed F-value of 26.84 (*p* < 0.0001) confirms the linear model’s validity. The F-value of 5.00 (*p* = 0.1062) indicates an insignificant lack of fit in relation to pure error; this finding provides evidence that the PS data fitted the proposed model. The statistical analysis revealed that both the ST (X_2_) and EA type (X_3_) have a significant impact on the size of FLB-SPLs (*p* = 0.0178 and <0.0001, respectively). Figure 3 illustrates the linear plots for the individual effects of the investigated variables on the PS.

Furthermore, analysis showed that the size of the vesicles markedly decreases as sonication time increase. This observation is in accordance with previous studies. A significant inverse relationship between the size of gammaoryzanol nanoparticles and the sonication time was observed by Ghaderi et al. [31]. In another study, Badr-Eldin et al. [32] reported a similar inverse relationship between simvastatin spanlastics size and sonication time. The revealed impact of sonication time on size could be explained by the cavitation (pressure) energies produced from ultrasonication waves that pass through the colloidal dispersion system of the formulation. Such energies could result in particle fractionation, with consequent size reduction [33].

Regarding the EA type, the size of SPLs followed the order PVA > SDC > Tween 80 > TPGS. This order can be attributed to the HLB values of the edge activators used. PVA has the highest HLB value of 18, followed by SDC (HLB = 16.7), Tween 80 (HLB = 15), and TPGS (HLB = 13.2). [34,35]. As reported previously, HLB value has a direct relationship with SPLs size. Increased particle sizes with elevated HLB value surfactants could be due to higher surface energy and water uptake of these surfactants with higher HLB values [36].

### 3.4. Statistical Analysis of the Influence of Variables on ZP (Y_2_)

ZP, which is related to the surface charge of the particles, is considered an important indicator of physical stability against aggregation. Nano-sized systems with ZP values greater than ±30 mV are considered stable, due to high electrostatic repulsion that guards against particles clumping [37]. The proposed SPLs were negatively charged with ZP, ranging from −24.82 ± 0.54 to −36.4 ± 1.2, as demonstrated in Table 2. The negative charge of the SPLs could be attributed to the partially negative groups existing in the polar head of Span. The direction of such polar heads to the external aqueous phase could imply a net negative charge for the developed SPLs [38]. The appropriateness of the linear model was confirmed by the computed F-value of 55.17 (*p* < 0.0001) in the ANOVA analysis (Table 4). The F-value of 2.78 (*p* = 0.2168) indicates an insignificant lack of fit in relation to pure error, providing further evidence for the fitting of the ZP data to the suggested model. The statistical analysis revealed that both Span: EA (X_1_) and EA type (X_3_) have a significant impact on the ZP of FLB-SPLs (*p* = 0.0319 and <0.0001, respectively). As per the higher *p*-value, it is evident that EA type effect was more pronounced than the Span to EA ratio.

**Figure 4:**
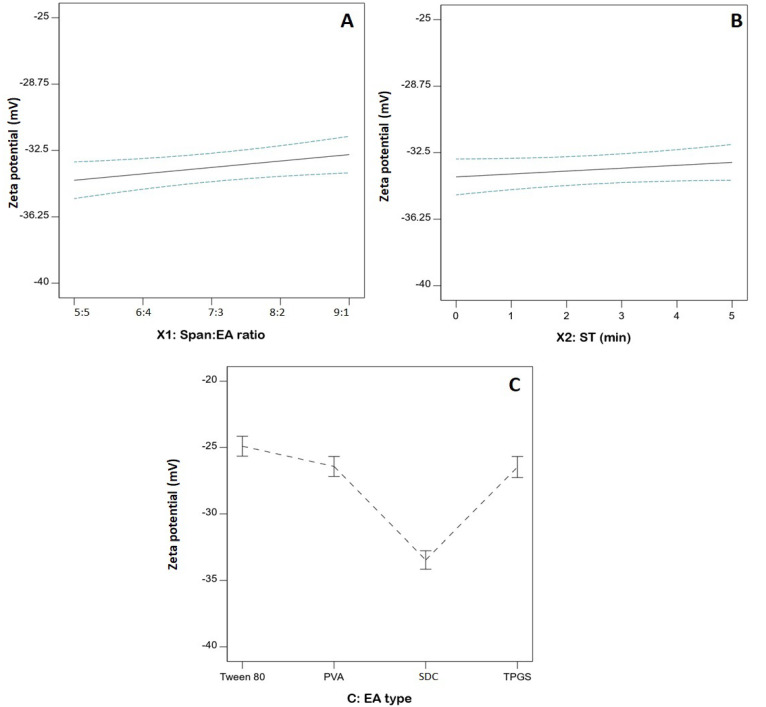
Linear plots showing individual effects on FLB-SPLs’ zeta potential. (**A**) Span: EA ratio, (**B**) ST; (**C**) EA type. Abbreviations: FLB, flibanserin; SPL, spanlastics; EA, edge activator; ST, sonication time; PVA, polyvinyl alcohol; SDC, sodium deoxycholate; TPGS, D-α-tocopheryl polyethylene glycol succinate.

Figure 4 illustrates the linear plots for the individual effects of the investigated variables on the ZP. Regarding the EA type, SPLs prepared using SDC had higher absolute ZP than those prepared using other surfactants. Furthermore, the effect of the Span: EA ratio was also mainly notable with SDC, as shown in Table 2, where the absolute ZP significantly increased with increasing amounts of edge activator. For the other edge activators, there was a negligible difference among different types and ratios. This observation could be due to the negative charge of SDC, contrary to the non-ionic nature of the other surfactants that were used.

### 3.5. Optimization of FLB-SPLs

**Figure 5:**
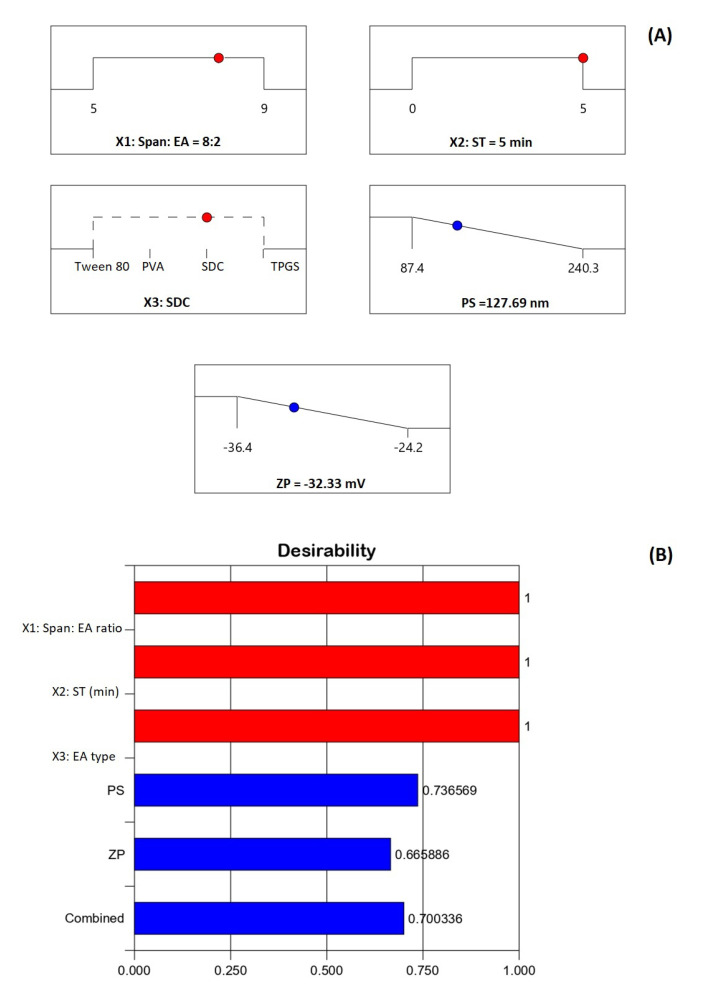
(**A**) Ramp graphs for the optimized variable levels and predicted responses of the optimized FLB-SPLs; (**B**) bar graph for the desirability of the optimization process. Abbreviations: FLB, flibanserin; SPL, spanlastics; EA, edge activator; ST, sonication time; PVA, polyvinyl alcohol; SDC, sodium deoxycholate; TPGS, D-α-tocopheryl polyethylene glycol succinate; PS, particle size; ZP, zeta potential.

Pharmaceutical optimization mainly aims to predict the levels of variables that will generate a formulation with the anticipated characteristics. This study adopted numerical optimization to develop SPLs with minimized size and simultaneous maximized absolute ZP value. The software projected the levels of independent variables that could attain the required objectives with the greatest desirability when all the variables combined. The ramp graphs shown in Figure 5A present the optimized levels and the predicted responses, while the desirability values are shown in Figure 5B. The overall desirability of the predicted combination of variables to achieve the desired goals of the responses is 0.70036. The measured responses were 129.70 nm for size and −33.17 for ZP, highlighting good permeation and high stability against aggregation. The predicted responses correlated well with the observed ones, with low percentage relative error rates of 1.54% and 2.53% for PS and ZP, respectively. This comparatively low error percentage affirms the credibility of the optimization process. It is noteworthy that, although positively charged particles are reported to have better brain permeation compared to negatively charged ones, previous studies have also reported appropriate brain delivery for nano-sized formulations with negative surface charge [39].

### 3.6. Characterization of Optimized FLB-SPLs

**Figure 6:**
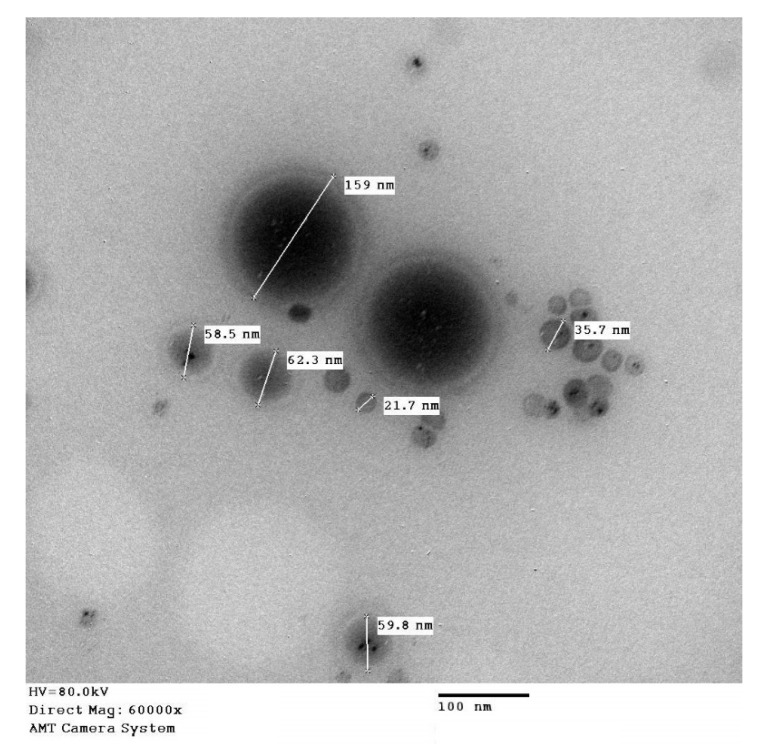
TEM image of optimized FLB-SPLs.

The characterization of optimized FLB-SPLs for EE% revealed the percentage of FLB was (80.4 ± 6.8%) entrapped in the optimized formula. In addition, the optimized FLB-SPLs TEM image shown in Figure 6 features separate spherical nanostructures (no aggregation) with globule diameters ranging from 21.7 nm to 159 nm, as indicated in the figure. The technique used in measuring size in the TEM application is different from the DLS technique, and the variation in the size data could be attributed to the sample preparation for TEM investigation (drying and staining), which could possibly affect the size distribution. Additionally, size measured by the z average mean size (the DLS technique used in particle size determination) is sensitive to minor changes in the sample.

### 3.7. In Vivo Pharmacokinetic Assessment of Optimized FLB-SPLs

**Figure 7:**
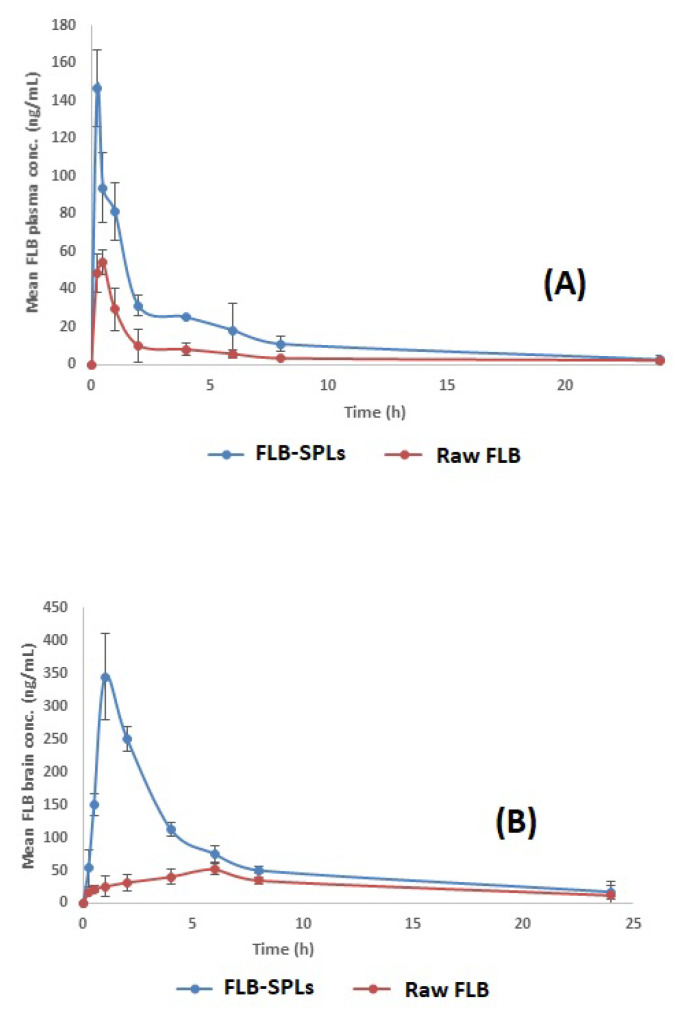
Mean (**A**) plasma concentrations; (**B**) brain concentrations vs. time of FLB after nasal administration of optimized FLB-SPLs compared to raw FLB in rats at a dose of 10 mg/kg. (Results presented as mean ± SD, n = 3). Abbreviations: FLB, flibanserin; SPL, spanlastics.

The average FLB concentration in rats’ plasma and brains following the intranasal administration of raw FLB powder and optimized FLB-SPLs is shown in Figure 6A,B and Figure 7A,B, respectively. The calculated pharmacokinetics parameters are shown in Table 5. The optimized FLB-SPLs showed a significantly higher C_max_, AUC_0–24_, and AUC_0–∞_ in both plasma and brain (*p* < 0.05) in comparison to raw FLB administered intranasally. On the other hand, the optimized formulation showed 2.11- and 2.23-fold increases in bioavailability for plasma and brain (respectively), indicating the higher abundance of the drug in plasma and in the target organ (brain) in comparison to raw FLB powder, with a significantly lower T_max_. Lowering the time required to reach the maximum drug concentration (T_max_) indicates faster drug absorption into both the systemic circulation and the brain tissues.

Considerable research has focused on intranasal administration for systemic medication delivery [40,41]. Intranasal drug delivery is considered a promising route for drug administration because of the relatively high permeability of the nasal epithelium, its high vascularization in the lamina propria, and because it allows for the avoidance of the hepatic first-pass metabolism [42,43]. The improved C_max_, AUC_0–24_, and AUC_0–∞_ in both the plasma and brain data for optimized FLB-SPLs, compared with raw FLB, could be attributed to the nanosized FLB distribution within the SPLs, which leads to enhanced permeation and absorption when compared to the higher particle size (possibly > 200 µm) for raw FLB. Furthermore, the relatively short T_max_ attained after intransal administration could be credited to the possible rapid absorption via the nasal mucosa directly into the blood stream [44]. It should be noted that the improved plasma FLB data for the optimized formula could also be partially related to the FLB’s gastrointestinal absorption, as a result of swallowing the formula after intranasal administration [45].

## 4. Conclusions

Numerical optimization was successfully employed to optimize FLB-SPLs with a minimized size of 129.70 nm and a zeta potential of −33.17 mV. When the results from intra-nasal dosing in rats were analyzed, the optimized spanlastics formulation showed 2.11- and 2.23-fold enhanced bioavailability in the plasma and the brain, respectively, compared to raw FLB. Less time is required to reach the maximum drug concentration, indicating faster drug absorption into both the systemic circulation and brain tissues. The findings of the study highlight the potential of the proposed spanlastics as an efficient drug carrier for the trans-nasal delivery of drugs to the brain.

## Figures and Tables

**Table 1:** Levels of independent variables and desirable constraints of the responses for the response-surface randomized D-optimal design employed for FLB-SPLs’ formulations.

Independent Variables	Levels			
X_1_: Span: edge activator ratio (*w*/*w*)	5:5	7:3	9:1	
X_2_: Sonication time (min)	0.0	2.5	5.0	
X_3_: Edge activator type	Tween 80	PVA	SDC	TPGS
Responses	Desirability constraint			
Y_1_: Particle size (nm)	Minimize			
Y_2_: Absolute zeta potential (mV)	Maximize			

Abbreviations: FLB, flibanserin; SPL, spanlastics; PVA, polyvinyl alcohol; SDC, sodium deoxycholate; TPGS, D-α-tocopheryl polyethylene glycol succinate.

**Table 2:** Composition of FLB-SPLs’ experimental runs, prepared according to the response-surface randomized D-optimal design, and their relative responses.

Run No.	Independent Variables			Average Responses ± SD *	
	X_1_	X_2_	X_3_	Y_1_	Y_2_
1	7:3	2.5	SDC	143.8 ± 4.9	−32.9 ± 1.1
2	9:1	5.0	SDC	131.6 ± 2.7	−31.4 ± 1.7
3	7:3	5.0	Tween 80	107.6 ± 2.1	−24.9 ± 0.7
4	5:5	2.5	TPGS	87.4 ± 1.9	−26.4 ± 0.9
5	5:5	0.0	SDC	194.8 ± 3.3	−36.4 ± 1.2
6	7:3	2.5	SDC	156.7 ± 3.7	−33.1 ± 1.4
7	9:1	2.5	PVA	240.3 ± 6.9	−26.1 ± 0.9
8	7:3	0.0	PVA	231.8 ± 7.1	−26.9 ± 1.1
9	7:3	2.5	SDC	162.1 ± 5.3	−34.2 ± 1.6
10	5:5	0.0	Tween 80	123.4 ± 4.2	−25.7 ± 0.6
11	7:3	5.0	PVA	201.5 ± 5.8	−27.1± 0.5
12	9:1	0.0	Tween 80	139.8 ± 3.8	−24.8 ± 0.5
13	5:5	2.5	PVA	205.0 ± 5.9	−25.6 ± 0.9
14	7:3	5.0	Tween 80	111.5 ± 2.4	−24.2 ± 0.4
15	9:1	5.0	TPGS	89.7 ± 2.1	−25.8 ± 0.6
16	9:1	0.0	SDC	154.9 ± 3.9	−32.1 ± 0.9
17	5:5	5.0	SDC	172.1 ± 4.1	−34.1 ± 1.3
18	9:1	0.0	TPGS	118.4 ± 3.5	−26.5 ± 0.8

Abbreviations: FLB, flibanserin; SPL, spanlastics; X_1_: Span: edge activator ratio; X_2_: sonication time (min); X_3_: edge activator type; PVA, polyvinyl alcohol; SDC, sodium deoxycholate; TPGS, D-α-tocopheryl polyethylene glycol succinate; Y_1_; particle size (nm); Y_2_, zeta potential (mV); SD, standard deviation. * Results are presented as mean ± standard deviation (*n* = 5).

**Table 3:** ANOVA of particle size of FLB-SPLs.

Source	Sum of Squares	Degrees of Freedom	Mean Square	F-Value	*p*-Value
Model	33,012.17	5	6602.43	26.84	<0.0001
X_1_: Span: EA	3.96	1	3.96	0.0161	0.9011
X_2_: ST (min)	1852.57	1	1852.57	7.53	0.0178
X_3_: EA type	30,808.12	3	10,269.37	41.75	<0.0001
Residual	2951.98	12	246.00		
Lack of fit	2767.56	9	307.51	5.00	0.1062
Pure error	184.42	3	61.47		
Cor total	35,964.15	17			

Abbreviations: FLB, flibanserin; SPL, spanlastics; EA, edge activator; ST, sonication time.

**Table 4:** ANOVA of zeta potential of FLB-SPLs.

Source	Sum of Squares	Degrees of Freedom	Mean Square	F-Value	*p*-Value
Model	262.59	5	52.52	55.17	<0.0001
X_1_: Span: EA	5.61	1	5.61	5.89	0.0319
X_2_: ST (min)	2.00	1	2.00	2.10	0.1728
X_3_: EA type	250.92	3	83.64	87.86	<0.0001
Residual	11.42	12	0.9520		
Lack of fit	10.20	9	1.13	2.78	0.2168
Pure error	1.23	3	0.4083		
Cor total	274.02	17			

Abbreviations: ANOVA, analysis of variance; FLB, flibanserin; SPL, spanlastics; EA, edge activator; ST, sonication time.

**Table 5:** In vivo pharmacokinetic parameters following the intra-nasal administration of optimized FLB-SPLs compared to raw FLB.

Pharmacokinetic Parameter	Plasma Data		Brain Data	
	Raw FLB	FLB-SPL	Raw FLB	FLB-SPL
C_max_ (ng/mL)	54.37 ± 6.56	147.08 ± 24.04	51.91 ± 8.31	345.02 ± 65.15
T_max_ (h)	0.5	0.25	6	1
AUC_0–24_ (ng·h/mL)	145.04 ± 23.82	387.16 ± 38.90	671.46 ± 36.17	1673.2 ± 125.28
AUC_0–∞_ (ng·h/mL)	195.39 ± 25.64	413.29 ± 40.12	849.95 ± 40.27	1901.22 ± 130.16
Relative bioavailability	---	211.52%	---	223.68%

Abbreviations: FLB, flibanserin; SPL, spanlastics.

## Data Availability

Data are contained in the article.
